# A prospective, single-arm trial of fluorescent ureteroscopy-assisted thulium-holmium:YAG dual laser ablation for upper urinary tract cancer: Study protocol of the FLUAM trial

**DOI:** 10.1016/j.conctc.2022.100902

**Published:** 2022-03-02

**Authors:** Makito Miyake, Takashi Yoshida, Nobutaka Nishimura, Yuki Oda, Takuto Shimizu, Tomonori Nakahama, Shunta Hori, Yosuke Morizawa, Daisuke Gotoh, Yasushi Nakai, Satoshi Anai, Kazumasa Torimoto, Tomomi Fujii, Nobumichi Tanaka, Kiyohide Fujimoto

**Affiliations:** a Department of Urology, Nara Medical University, Kashihara, Nara, Japan; b Department of Urology and Andrology, Kansai Medical University Hospital, Hirakata, Japan; c Department of Urology, Hirao Hospital, Kashihara, Nara, Japan; d Department of Diagnostic Pathology, Nara Medical University, Kashihara, Nara, Japan; e Department of Prostate Brachytherapy, Nara Medical University, Kashihara, Nara, Japan

**Keywords:** Upper urinary tract cancer, Transurethral surgery, 5-Aminolevulinic acid, Photodynamic diagnosis, UroVysion

## Abstract

**Background:**

Latest guidelines recommend kidney-sparing management as the primary treatment option for selected patients with upper urinary tract urothelial carcinoma (UTUC). One of the biggest issues of ureteroscopic laser ablation (ULA) is a high rate of surgical site recurrence, which is largely attributed to residual lesions at the initial ULA. Another clinical issue is a significant lack of non-invasive reliable detection tools of urinary recurrent tumors in this treatment setting.

**Methods:**

The FLUAM trial is a prospective, single-center, single-arm pilot trial to investigate the efficacy of 5-aminolevulinic acid-mediated photodynamic diagnosis (ALA-PDD)-assisted ULA for localized UTUC and the usefulness of the UroVysion® assay (multiprobe fluorescence in situ hybridization) as a monitoring test after the kidney-sparing treatment. After the screening and registration, a total of 20 patients with localized UTUC will undergo the initial ALA-PDD-assisted ULA followed by the second look ALA-PDD-assisted ureteroscopic examination. The primary endpoint is progression-free survival. Secondary endpoints include patient reported outcomes, diagnostic accuracy of UroVysion assay to detect tumor recurrence, adverse events, and safety of the intervention.

**Conclusion:**

The goal of this trial is to determine the potential benefit of ALA-PDD assistance in patients who undergo the ULA. The evidence of this novel technique is still limited. The results are expected to change the standard of care and lead to better management of localized UTUC.

**Trial registration:**

This clinical trial was prospectively registered with the Japan Registry of Clinical Trials on 23 June 2021. The reference number is jRCTs051210042, nara0023 (Certified Review Board of Nara Medical University).

## 1 Introduction

Upper urinary tract urothelial carcinoma (UTUC) is relatively uncommon, accounting for only 5%–10% of all new cases of UC [1]. UTUC presents unique approaches for diagnosis, surgical treatment, and pre-/post-operative management. Radical nephroureterectomy (RNU) with removal of distal ureter (bladder cuff excision) is the gold standard for localized UTUC [[1], [2], [3]], but is associated with a significant loss of renal function [4,5]. Thus, kidney-sparing management involves alternatives that do not compromise oncological outcomes in well-selected patients [2,6,7]. The latest European Association of Urology guidelines on UTUC lists three recommendations as follows [2]:

i) offer kidney-sparing management as primary treatment option to patients with low-risk tumors (strongly recommended),
ii) offer kidney-sparing management (distal ureterectomy) to patients with high-risk tumors limited to the distal ureter (poorly recommended), and
iii) offer kidney-sparing management to patients with solitary kidney and/or impaired renal function, providing that it will not compromise survival (strongly recommended).

Intraluminal ureteroscopic laser ablation (ULA) has provided acceptable oncological and functional outcomes, especially for patients with low-grade (LG)/low-volume tumors in the last two decades [[8], [9], [10], [11], [12]]. The recent advancement in visual technologies, flexible ureteroscopies, and laser energy equipment have made it more practical. Two reports demonstrated the safety and efficacy of thulium (Tm)-holmium (Ho): yttrium aluminum garnet (YAG) dual laser ablation [8,9]. The combination of Tm:YAG laser with continuous wave and Ho:YAG laser with long pulse mode enables efficient tumor coagulation, vaporization, and resection under comfortable visual control. One of the biggest concerns of ULA is high rate of surgical site recurrence, reportedly ranging from 30.7% to 90.5% [[9], [10], [11], [12], [13]], which is largely attributed to residual lesions at the initial ULA. Previous reports demonstrated the advantage of oral 5-aminolevulinic acid-mediated photodynamic diagnosis (ALA-PDD) using fluorescent ureteroscopy to detect invisible lesions, with conventional white light endoscopy [[14], [15], [16]]. Our primary interest in this clinical trial was whether the use of PDD technology provides decreased surgical site recurrence and subsequent progression, through complete resection of UTUC.

The patients should be followed up closely in kidney-sparing management. Ureteroscopy with or without biopsy, cystoscopy, urinary cytology, and chest/abdominal/pelvic computed tomography (CT) or magnetic resonance imaging (MRI) are performed at regular intervals for surveillance [2,8]. Unfortunately, there is a significant lack of non-invasive reliable detection tools of urinary recurrent tumors in this treatment setting. Previous studies suggested that the UroVysion® test was a useful tool for the diagnosis not only of primary UTUC but recurrent UTUC after kidney-sparing surgery [17,18].

This prospective, single-center, single-arm pilot trial will investigate the efficacy and safety of ALA-PDD-assisted ULA for localized UTUC and the feasibility of UroVysion test combined with conventional urinary cytology as a detection marker of recurrent UTUC after the kidney-sparing surgery.

## 2 Protocol digest of the FLUAM trial

### 2.1 Study type and ethical issues

This clinical trial is a prospective, single-center, single-arm trial for patients with localized UTUC, which is ongoing at Nara Medical University Hospital. This trial complied with the Declaration of Helsinki regarding investigation in humans. Its ethical clearance and the final study protocol (version 1.0 on June 21st, 2021), including the subject information, informed consent forms, and associated documents, were approved by the Certified Review Board of Nara Medical University (institution ID: CRB5200002). Informed consent and written consent forms of patients are mandatory before study participation. This clinical trial was prospectively registered with the Japan Registry of Clinical Trials on June 28th, 2021 (the reference number: jRCTs051210042). The URL of trial registry record is found in https://jrct.niph.go.jp/latest-detail/jRCTs051210042.

### 2.2 Inclusion criteria

**Fig. 1:**
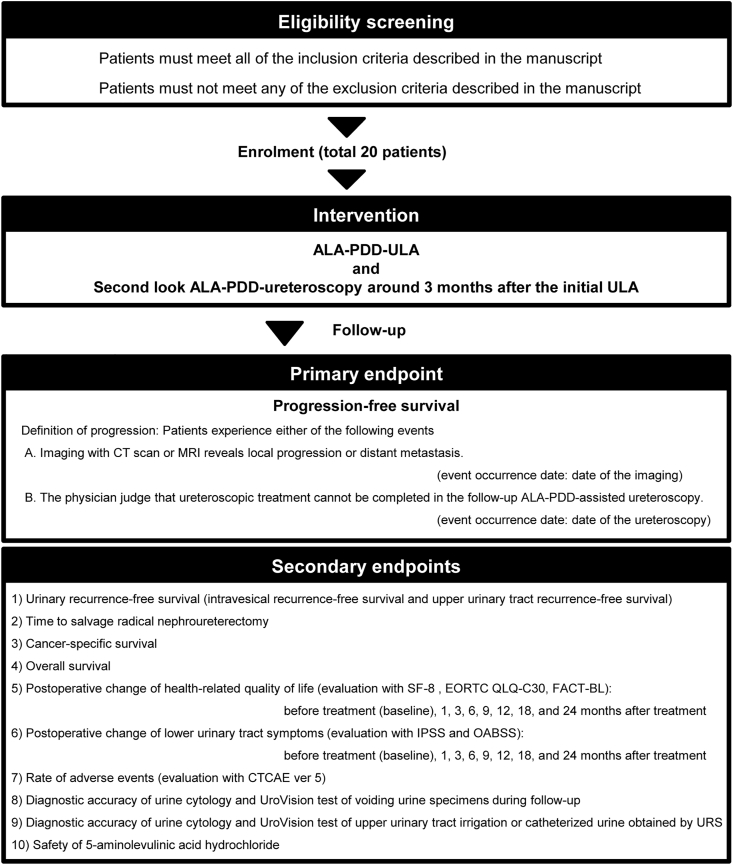
**The flow chart, patient enrolment, endpoints of the FLUAM trial.** Patients must meet all the inclusion criteria to be eligible for this trial.

The flow chart, patient enrolment, endpoints of the FLUAM trial are depicted in Fig. 1. The trial design and protocol adhere to the Recommendations for Interventional Trials (SPIRIT) criteria [19]. The completed SPIRIT checklist can be found in Supplementary data. Investigators and patients will be aware of the intervention of oral administration of ALA. Patients will undergo a general work-up and radiographical examinations, including CT, CT-urography (CT-U) or MRI, cystoscopy, and urine cytology. Diagnostic ureteroscopy with biopsy ± upper urinary tract catheterization or irrigation cytology are performed to evaluate the tumor grade, tumor location, architecture, and feasibility of the ULA. Tumors are staged and graded according to the eighth edition of the American Joint Committee on Cancer (AJCC) TNM classification [20], by an experienced uropathologist (T. Fujii). Prior to enrolment in this trial, patients must meet all of the following inclusion criteria:

(i) Patients with clinical Ta–1N0M0 localized UTUC desire kidney-preservation therapy and meet any of the following A to E.

A. Tumor develops in patients with solitary kidney or tumor develops bilaterally.
B. Patients with chronic kidney disease are expected to avoid renal replacement therapy in the future by kidney-preservation therapy.
C. Due to poor performance status, it is clinically judged that total RNU is not indicated.
D. Patients have a single tumor with a major axis of 10 mm or less and have a healthy kidney on the contralateral side.
E. Patients desire kidney-preservation therapy, although they are recommended RNU.

(ii) Age, 20 years or older (regardless of gender)
(iii) The pathological diagnosis is confirmed by ureteroscopic biopsy.
(iv) The name and disease status have been informed to the patient, and he/she must be given an explanation when participating in this study. The written consent at his/her must be obtained with sufficient understanding.
(v) Bone marrow function and organ function (excluding renal function) are sufficient by the following laboratory findings within 60 days:

A. Hemoglobin ≥9.0 g/dL (no transfusion within 4 weeks)
B. White blood cell count, the lower limit to 12,000/mm^3^
C. Neutrophil count ≥2,000/mm^3^
D. Platelet count ≥100,000/mm^3^
E. Total bilirubin ≤2 × the upper limit of normal
F. Aspartate aminotransferase (AST) ≤ 2 × the upper limit of normal
G. Alanine aminotransferase (ALT) ≤ 2 × the upper limit of normal

### 2.3 Exclusion criteria

Prior to enrolment in this trial, patients must not meet any of the following exclusion criteria:

(i) Patients with a history of allergic reaction to ALA.
(ii) Pregnant women, lactating women, and patients who may be pregnant.
(iii) Patients with liver dysfunction and inflammatory disease.
(iv) Patients with porphyria or patients with a history of allergic reaction to porphyrin-related substances.
(v) Patients taking concomitantly prohibited drugs and foods, which are known to cause photosensitivity (skin reaction): Tetracycline antibiotics, sulfonamides, new quinolone antibacterial agents, hypericin (Hypericum erectum extract), etc., Hypericum erectum (St. Jones Wort)
(vi) Cases in which patients cannot give consent or take the medicine on their own initiative.
(vii) Deemed inappropriate for enrolment as per the judgment of the physician.

### 2.4 Preoperative procedure and surgical device

Approximately 3 h (range, 2–4 h) prior to ULA, patients received by mouth the water-dissolved ALA hydrochloride solution (SBI Pharmaceuticals, Tokyo, Japan) at a dose of 20 mg/kg body, which is the same dose as the current clinical practice for non-muscle invasive bladder cancer in Japan [21].

The ULA is performed under general or spinal anesthesia in the lithotomy position. Tumor visualization and PDD assistance was carried out using the Storz D-LIGHT System (KARL STORZ GmbH & Co. KG; Tuttlingen, Germany) and the Storz Professional Image Enhancement System (IMAGE1S™, KARL STORZ). Rigid 7-Fr Ureto-Renoscopes, flexible Ureto-Fiberscopes Flex-X^2^, and an eyepiece filter specific for detection of protoporphyrin IX (KARL STORZ) were used. We can switch imaging modes of white light and blue light frequently during the operation to set the resection margin and detect the residual lesion. A UROMAT E.A.S.I. (KARL STORZ) is used to keep appropriate pressure levels to obtain an adequate irrigation flow during the ULA, especially for renal pelvic tumors. A Revolix 120 Tm:YAG laser system and Sphinx jr. Ho:YAG laser system (LISA Laser Products OHG, Katlenburg-Lindau, Germany) are used for tumor laser ablation. For both laser systems, 272-mm or 550-mm laser fibers are used depending on the tumor location and the use of flexible ureteroscope or rigid ureteroscope.

### 2.5 Surgical procedure of ALA-PDD-ULA

**Fig. 2:**
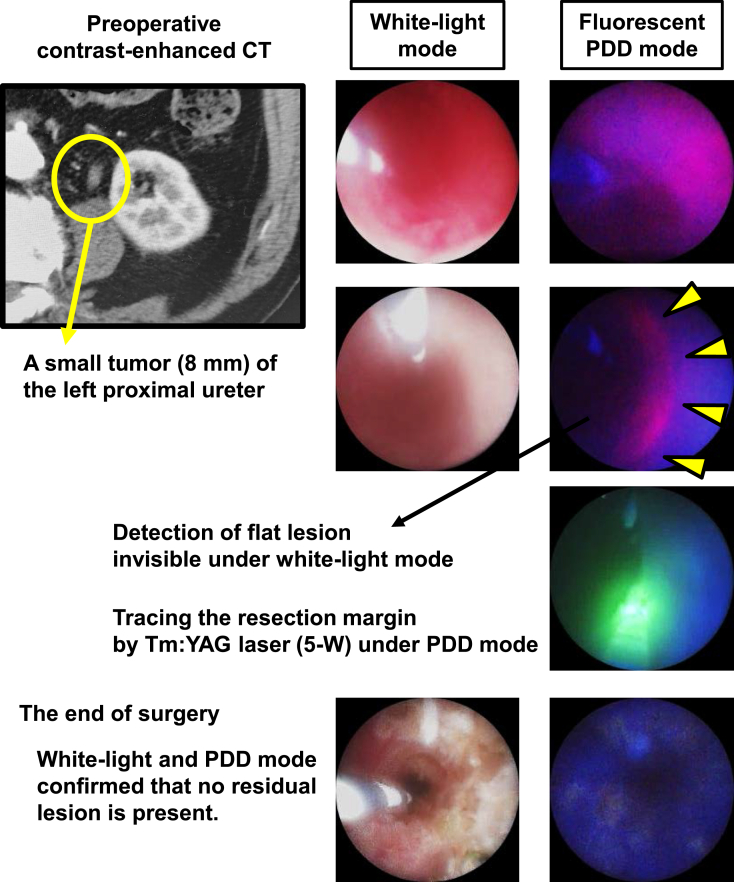
**A representative case showing benefits of using photodynamic diagnosis (PDD) assistance during the ureteroscopic laser ablation (ULA).** A 74-year-old man presented to a urology clinic with gross hematuria. A contrast-enhanced CT revealed an 8-mm tumor of the left proximal ureter. The ureteroscopic biopsy pathologically confirmed low-grade urothelial carcinoma. The patient was considered to be well indicated for the ALA-PDD-ULA. First, a papillary-shaped tumor is observed under white-light mode. Then, the PDD mode detects a spreading flat lesion which is invisible under white-light mode. We trace the resection margin by Tm:YAG laser (setting: 5-W) under PDD mode. At the end of surgery, white-light and PDD mode confirmed that no residual lesion is present.

Resection and ablation of all the visible tumors is performed according to the surgical method as previously described [8]. A ureteral access sheath (10/12-Fr to 12/14-Fr) is placed depending on the ureteral condition, and a flexible ureteroscope is used for proximal ureteral tumors or renal pelvic tumors, while a rigid ureteroscope is used for distal or middle ureteral tumors. Tumors are first observed with caution under white light mode and PDD mode to determine resection margin. Then, vaporization and coagulation by Tm: YAG laser are performed. Ablation and resection by Ho:YAG laser can be added on a case-by-case basis after the tumor becomes ischemic. Large tumor fragments are retrieved using a stone extraction basket. Lastly, observation under the PDD mode is performed to detect residual lesion and, when present, the tumors are additionally ablated with the Tm:YAG and Ho:YAG lasers until all PDD-positive tumors are eradicated. A representative case treated with ALA-PDD-ULA is shown in Fig. 2. A 6-Fr double J-stent ureteral stent and a urethral catheter were inserted at the end of surgery. All patients were instructed to avoid exposure to sunlight or bright indoor light for up to 48 h after oral administration of ALA.

### 2.6 Follow-up schedule and data collection

**Fig. 3:**
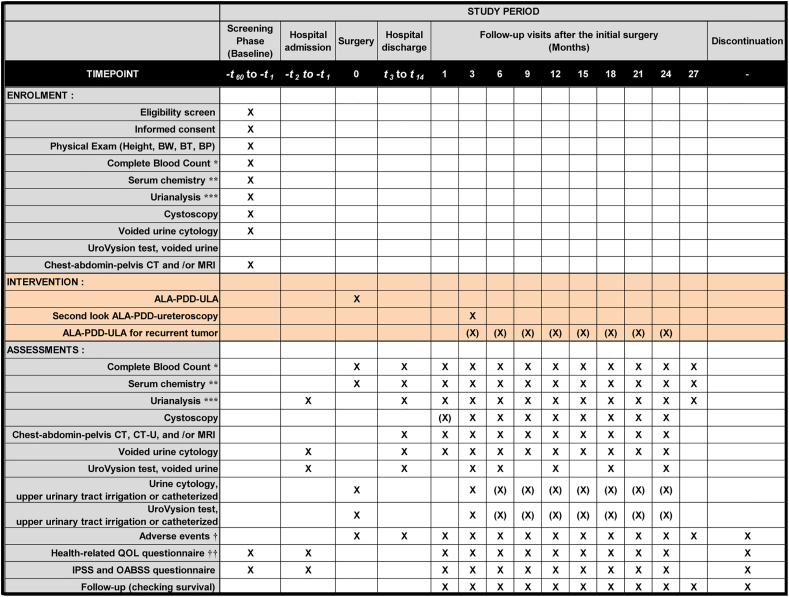
Intervention and assessment schedule of the FLUAM trial. Follow-up visits and data collection should occur approximately 1, 3, 6, 9, 12, 15, 18, 21, 24, and 27 months from the initial surgery. Patients will complete a set of questionnaires at every visit, and follow-up information may be collected via medical charts. The Case Report Form will include information regarding past history, concomitant medications, and any medications taken after the treatment. Chest–abdomen–pelvis computed tomography (CT) and/or magnetic resonance imaging (MRI) should be performed for TNM classification. X, mandatory; (X), optional, ALA-PDD-ULA after 6 months is performed when the radiographic examination suspect tumor recurrence in the upper urinary tract. *hemoglobin, hematocrit, white blood cell count and fractions, platelet count; **aspartate transaminase (AST), alanine transaminase (ALT), γ-glutamyl transpeptidase (γ-GTP), total bilirubin, alkaline phosphatase (ALP), lactate dehydrogenase (LDH), total protein, albumin, serum creatinine, blood urea nitrogen, uric acid, total cholesterol, triglyceride, C-reactive protein (CRP), sodium, potassium, chloride; ***Urine dipstick test (specific gravity, pH, protein, glucose, bilirubin, urobilinogen, ketone body, and occult blood) and urine sediment test; †according to the Common Toxicity Criteria for Adverse Events (CTCAE v 5.0) translated into Japanese; †† questionnaires SF-8™, EORTC QLQ-C30, and FACT-BL. Abbreviations: BW, body weight; BT, body temperature; BP, blood pressure; ALA, 5-aminolevulinic acid; PDD, photodynamic diagnosis; ULA, ureteroscopy laser ablation; QOL, quality of life; IPSS, International Prostate Symptom Score; OABSS, overactive bladder symptom score.

Patient data will be collected before and after the surgery; 1, 3, 6, 9, 12, 15, 18, 21, 24, and 27 months after the initial ALA-PDD-ULA; and at the end of the trial (Fig. 3). To improve monitoring adherence, the clinician will take time to explain the need for post-operative surveillance examination, including second-look URS, and encourage the participants to undergo routine cystoscopy, urine cytology, and CT/CT-U/MRI. The assessment includes patient-reported outcomes, such as the SF-8™, EORTC QLQ-C30, FACT-BL, International Prostate Symptom Score (IPSS), and overactive bladder symptom score (OABSS). The postoperative observational evaluation such as PROs can be discontinued upon participant request. The data will be documented in specific Case Report Forms for complete blood count, serum chemistry, cystoscopy, urine cytology, CT/CT-U/MRI, genitourinary adverse events (AEs), and other possible AEs using the Common Toxicity Criteria for Adverse Events (CTCAE v 5.0). When recurrence is suspected in imaging examinations, ALA-PDD-ureteroscopy is considered and, if recurrent tumor is present, ALA-PDD-ULA is additionally performed. AEs strongly related to the intervention of ALA-PDD-ULA are listed as follows: “Intraoperative urinary injury,” “Urinary tract infection,” “Fever,” “Renal hemorrhage,” “Hematuria,” “Creatinine increased”, “Urinary frequency”, “Photosensitivity”, “Hepatobiliary disorders”, “Urinary tract pain”, “Urinary urgency”, and “Dysuria”. Any other potential AEs are recorded and grouped into ‘related AEs’ or ‘unrelated AEs.’ When serious AEs occur, investigators report to the Safety Monitoring Committee.

To protect the patient's personal information, unique identification codes (FLUAM IDs) will be assigned to all patients. All the data will be protected in password-accessible electronic data files on secure servers before, during, and after the trial, and only investigators will be able to access the files. All data and documents will be deleted and discarded five years after the trial ends, unless the data are being used for another study.

### 2.7 Sample size setting

The primary endpoint of this trial is progression-free survival (PFS). Progression is noted when patients experience either of the following events: A) Imaging with CT scan or MRI reveals local progression or distant metastasis (event occurrence date: date of the imaging) and B) The physician judges that ureteroscopic treatment cannot be completed in the follow-up ALA-PDD-assisted ureteroscopy (event occurrence date: date of the ureteroscopy). The date of the initial ALA-PDD-ULA is taken as the starting point. The historical data of the Department of Urology and Andrology, Kansai Medical University Hospital was obtained from consecutive patients undergoing ULA without PDD assistance. The 2-year progression-free survival (PFS) rate of ULA without PDD assistance and that with PDD assistance was 58% and 90%, respectively. The required sample size was determined to test our null hypothesis of superiority in the 2-year PFS rate after the initial surgery. Assuming that the threshold survival rate is 58% and expected survival rate is 90%, 14 patients are required with a 3-year registration period and 2-year tracking period years to provide 90% power (β = 0.10) and an α level of 0.05 (two-sided). Given the 20–30% proportion of ineligible patients, a total of 20 trial cases will be enroled in this study. We calculated the sample size using Statistical Tools 2019 provided by Cancer Research and Biostatistics (https://stattools.crab.org/).

### 2.8 Interim analysis and monitoring

Because this is a short-term, single-arm, pilot study, we will not conduct interim analysis for the clinical efficacy of ALA-PDD-ULA. However, the safety of interventions will be independently evaluated by the Data and Safety Monitoring Committee at the time as follows:

i) Critical modification of the study protocol is required
ii) Any serious adverse event associated with this agent occurs
iii) A critical problem is observed while monitoring
iv) The principal investigator needs the judgment of this committee

Moreover, the monitoring committee independently evaluates whether the study is implemented in compliance with the study protocol, and the data are appropriately corrected according to a pre-arranged monitoring plan at the time when the first patients have been enroled and once per year.

### 2.9 Protocol of Fluorescence In Situ Hybridization (FISH) assay

As exploratory research, we evaluate the diagnostic accuracy of UroVysion test of voiding urine specimens during follow-up and upper urinary tract irrigation or catheterized urine obtained by URS. Sample preparation is performed according to the protocol for UroVysion® FISH bladder cancer assay (SRL, Inc., Tokyo, Japan). We transfer urine sediments into ThinPrep® PresevCyt® Solution containing 55% methanol (UV7) and subject fixed samples to the SRL laboratory. Trained observers who are blinded for clinicopathologic data assess enumeration of FISH signals. This assay enables the visualization of molecular alterations, including aneuploidy of chromosomes 3 (red), 7 (green), and 17 (aqua) and loss of locus 9p21 (gold), which are frequently seen in human bladder cancer. Specimens are considered to be positive for UroVysion test if four cells or more have a gain of 2 or more chromosomes (3, 7, or 17) or 12 cells or more have a loss of both copies of locus 9p21 among observed 25 morphologically abnormal cells [18,22].

### 2.10 Statistical analysis

Descriptive statistics will be computed for all study variables. EZR 1.54 (Saitama Medical Center, Jichi Medical University, 2020) and PRISM software version 7.00 (GraphPad Software, Inc., San Diego, CA, USA) were used for statistical analyses and data plotting, respectively. A two-sided P value of less than 0.05 will be considered to be statistically significant.

The full analysis set population and the per protocol set population will be separately analyzed for both the primary endpoint (two-year PFS rate) and survival outcomes listed in the secondary endpoints (Fig. 1). The per-protocol set population will be analyzed for the other secondary endpoints. The PFS of study population will be estimated by Kaplan Meier method, and 95% confidence interval (CI) will be calculated using Greenwood's formula for variance. When the lower limit of 95%CI of two-year PFS rate is more than 58% (threshold survival rate), the ALA-PDD-ULA is considered superior to the conventional ULA without PDD assistance. When the lower limit of 95%CI of two-year PFS rate is 58% or less, the ALA-PDD-ULA is considered not superior to the conventional ULA without PDD assistance. In addition, a sensitivity analysis of the primary endpoint will be performed using the Fine-Gray subdistribution hazard regression. This model considers patients who die of other causes (competing events) before recurrence or progression.

The amount of change in other post-operative observations over time will be analyzed using the repeated measures analysis of variance, Wilcoxon signed-rank sum test, or Mann-Whitney *U* test as appropriate. Mixed-effects models for repeated measures will be applied to detect multiple change-points in longitudinal data. To evaluate the diagnostic accuracy of UroVysion test, the result is compared to the pathological diagnosis obtained by URS biopsy. The sensitivity, specificity, positive predictive value, negative predictive value, and diagnostic accuracy are determined according to standard methods [23]. The sensitivities of UroVysion test and the combination with the conventional cytology are compared to that of conventional cytology alone using McNemar's test.

### 2.11 Dissemination

The results will be submitted to peer-reviewed journals for publication and presented at local and international scientific conferences. Also, the results will be made available to interested participants.

## 3 Conclusion

The goal of this trial is to determine the potential benefit of ALA-PDD assistance in patients who undergo the ULA. The evidence of this novel technique is still limited. The results are expected to change the standard of care and lead to better management of localized UTUC.

## Trial status

The study began in July 2021. Patient recruitment has not yet been completed, and the intervention program is ongoing. A follow-up and data collection will be completed in June 2026. The final results are expected in September 2026.

## Ethics approval and consent to participate

This clinical trial complied with the Declaration of Helsinki regarding investigation in humans. Its ethical clearance and protocol (version 1.0 on June 21st, 2021) including the subject information, informed consent forms, and associated documents, were approved by the Certified Review Board of Nara Medical University (institution ID: CRB5200002). Informed consent and written consent forms of patients are mandatory before study participation. This clinical trial was prospectively registered with the Japan Registry of Clinical Trials on June 28th, 2021 (the reference number: jRCTs051210042).

## Consent for publication

Not required.

## Availability of data and materials

The collected datasets used during this clinical trial are available from the corresponding author (M. Miyake) on reasonable request.

## Authors' contributions

MM is the principal investigator and conceived the study. TY is an advisor of the study. YM, DG, YN, SA, and KT participated in the design and intervention of the study. NN, YO, TS, TN, and NT are major contributors of the data acquisition. TF performs the histological examination of the prostate. SH will statistically analyze and interpreted the patient data regarding the adverse events and other associated outcomes. MM wrote the first draft of the manuscript and NT and KF substantively revised it. All authors provided input into the study design, provided intellectual input to the manuscript and approved the final version of the manuscript.

## Funding

No sponsor had the responsibility of initiating, managing, or financing the FLUAM trial. This trial is supported by the 10.13039/501100005782Clinical Research Promotion Program grant of Nara Medical University (Project ID, JHR2100001). SBI Pharmaceuticals Co., Ltd. provides 5-aminolevulinic acid hydrochloride to the investigators.

## Declaration of competing interest

M.M., S.H., D.G., Y.N, S.A., K.T., N.T., and K.F. receive 5-aminolevulinic acid hydrochloride from 10.13039/501100002363SBI Pharmaceuticals Co., Ltd. During the conduct of the study. M.M., S.A., K.T., N.T., and K.F. report receiving a joint research fund from SBI Pharmaceuticals Co., Ltd. outside the submitted work (the AMBER study; the Japan Registry of Clinical Trials ID jRCTs051190077). T.Y. received 5-aminolevulinic acid hydrochloride from SBI Pharmaceuticals Co., Ltd. outside the submitted work (the Japan Registry of Clinical Trials ID jRCTs051200004). N.N., Y.O., T.S., N.T., Y.M., and T.F. have no conflict of interest.

## List of abbreviations

UTUC: upper urinary tract urothelial carcinoma
RNU: radical nephroureterectomy
ULA: ureteroscopic laser ablation
LG: low-grade
Tm: thulium
Ho: holmium
TAG: yttrium aluminum garnet
ALA: 5-aminolevulinic acid
PDD: photodynamic diagnosis
CT: computed tomography
MRI: magnetic resonance imaging
CTCAE: Common Toxicity Criteria for Adverse Events
IPSS: International Prostate Symptom Score
OABSS: overactive bladder symptom score
FACT-BL: Functional Assessment of Cancer Therapy-Bladder
EORTC: The European Organization for Research and Treatment of Cancer QLQ-C30
AE: adverse event
PFS: progression-free survival
FISH: fluorescence in situ hybridization
